# Enteropathogenic Escherichia coli and Bacterial Overgrowth Co-infection Exacerbating Immune Checkpoint Inhibitor-Induced Colitis

**DOI:** 10.7759/cureus.103280

**Published:** 2026-02-09

**Authors:** Ermias A Kibru, Abdul-Rahaman A Ottun, John G Dusek, Eunice Hama, Bezawit M Fikadu

**Affiliations:** 1 Internal Medicine, Piedmont Athens Regional Hospital, Athens, USA; 2 Internal Medicine, Augusta University Medical College of Georgia, Athens, USA; 3 Internal Medicine, Adama General Hospital and Medical College, Adama, ETH

**Keywords:** bacterial overgrowth, checkpoint inhibitor colitis, electrolyte derangements, enteropathogenic escherichia coli, immune checkpoint inhibitors, immune-related adverse events, immunotherapy complications

## Abstract

Immune checkpoint inhibitor-induced colitis (ICIC) is a common gastrointestinal immune-related adverse event, particularly with combined ipilimumab-nivolumab therapy. We present a case of a 53-year-old man with metastatic mucosal melanoma receiving combination immunotherapy who developed severe diarrhea (>20 stools/day), profound hypokalemia (2.2 mmol/L), metabolic acidosis, and QTc prolongation. Multiplex stool PCR detected enteropathogenic *Escherichia coli* (EPEC), while colonoscopy with biopsy confirmed ICIC. Despite high-dose corticosteroids, the patient required >1,900 mEq potassium supplementation over 8 days. Stool studies revealed an osmotic gap of 123 mOsm/kg, and persistent bloating prompted empiric treatment for suspected small intestinal bacterial overgrowth (SIBO) with rifaximin. Clinical improvement occurred only after addressing all three pathologic processes: immune-mediated inflammation, EPEC infection, and bacterial overgrowth. This case demonstrates that enteric pathogens and bacterial overgrowth can significantly exacerbate ICIC, resulting in severe electrolyte derangements and prolonged clinical course. Clinicians should maintain high suspicion for co-infections in patients with ICIC who demonstrate disproportionate fluid and electrolyte losses or inadequate response to immunosuppression. Comprehensive diagnostic evaluation, including stool testing, endoscopic assessment, and physiologic profiling of diarrhea, enables targeted multimodal therapy beyond corticosteroid monotherapy.

## Introduction

Immune checkpoint inhibitors (ICIs) have revolutionized cancer treatment by restoring antitumor immune surveillance. However, blockade of cytotoxic T-lymphocyte antigen-4 (CTLA-4) and programmed cell death protein-1 (PD-1) pathways disrupts peripheral immune tolerance, resulting in immune-related adverse events (irAEs) affecting multiple organ systems [1,2].

The gastrointestinal tract is one of the most frequently affected sites, with combination ipilimumab-nivolumab therapy conferring the highest risk. Any-grade diarrhea occurs in approximately 40% of patients receiving dual therapy, while grade ≥3 colitis manifests in up to 17% [1,2]. Current management emphasizes prompt immunosuppression with corticosteroids, with escalation to biologic agents for refractory cases.

A critical yet often overlooked aspect of ICIC management is the potential for concurrent infectious etiologies that may amplify disease severity. Enteropathogenic *Escherichia (E.) coli* (EPEC), a non-Shiga toxin-producing pathogen, causes secretory diarrhea through intimate adherence to enterocytes and effacement of microvilli [3,4]. When EPEC infection occurs concurrently with ICIC, synergistic inflammatory and secretory mechanisms can result in profound fluid and electrolyte derangements exceeding those expected from either process alone.

We present a case demonstrating the clinical presentation of co-occurring ICIC, EPEC infection, and bacterial overgrowth, emphasizing the importance of comprehensive diagnostic evaluation and multimodal therapeutic approach.

## Case presentation

A 53-year-old man with right maxillary sinus mucosal melanoma presented to Piedmont Athens Regional Hospital with 7 days of high-volume watery diarrhea. He had recently completed his third cycle of combination immunotherapy with ipilimumab (1 mg/kg every 6 weeks) and nivolumab (3 mg/kg every 2 weeks). His symptoms included >10 non-bloody, non-mucoid watery stools per day, 5 episodes of non-bloody emesis daily, and mild generalized abdominal discomfort.

Emergency department assessment revealed a clinically dehydrated patient with stable vital signs. Abdominal examination demonstrated distension with mild diffuse tenderness. Laboratory investigations (Table 1) revealed marked abnormalities: white blood cell count 20.6 × 109/L, serum potassium 2.2 mmol/L, serum bicarbonate 12 mmol/L, creatinine 1.17 mg/dL (baseline 0.8 mg/dL), and a corrected QT interval of 568 ms. Multiplex gastrointestinal PCR detected enteropathogenic Escherichia coli (Table 2).

**Table 1:** Laboratory results at the time of presentation N/A^1^ - no reference range established by the lab N/A^2 ^- Fecal osmotic gap in mOsm/kg can be calculated using the following equation: Fecal Osmotic Gap = 290-2x((Na) + (K)). A fecal osmotic gap of 125 mOsm/kg or greater is indicative of osmotic diarrhea, whereas a result of 50 mOsm/kg or less indicates a secretory diarrhea.

Test type	Result	Reference range
White blood cell	20.60	4.00 - 10.50 x 10*3/µL
Neutrophils manual	80	40 - 75%
Bands manual	9	0 - 10%
Hemoglobin	15.0 g/dL	13.8 - 17.2 g/dL
Platelet	474	130 - 400 x 10*3/µL
Sodium	138 mmol/L	133 - 145 mmol/L
Potassium	2.2 mmol/L	3.3 - 5.1 mmol/L
Chloride	115 mmol/L	98 - 108 mmol/L
Bicarbonate	12 mmol/L	22 - 32 mmol/L
Magnesium	2.9 mg/dL	1.6 - 2.6 mg/dL
Calcium	9.8 mg/dL	8.4 - 10.2 mg/dL
Creatinine	1.17 mg/dL	0.50 - 1.20 mg/dL
Blood urea nitrogen	12 mg/dL	6 - 20 mg/dL
Alanine transaminase	31 U/L	7 - 52 U/L
Aspartate transaminase	20 U/L	12 - 50 U/L
Alkaline phosphatase	138 U/L	32 - 126 U/L
Total bilirubin	0.4 mg/dL	0.2 - 1.4 mg/dL
Stool osmolality	331 mOsm/kg	N/A^1^
Stool potassium	44.3 mEq/L	N/A^1,2^
Stool sodium	39 mEq/L	N/A^1,2^
Fecal leucocyte stain	Neg	Neg
Urine sodium	<10 mmol/L	N/A^1^
Urine potassium	14.4 mmol/L	N/A^1^
Urine creatinine	162.06 mg/dL	N/A^1^
Urine chloride	242 mmol/L	N/A^1^

**Table 2:** Multiplex GI panel by PCR

Component	Result
Campylobacter species	Not detected
Clostridium difficile toxin A/B	Not detected
Plesiomonas shigelloides	Not detected
Salmonella species	Not detected
Vibrio species	Not detected
Vibrio cholerae	Not detected
Yersinia enterocolitica	Not detected
Enteroaggregative E. coli (EAEC)	Not detected
Enteropathogenic E. coli (EPEC)	Detected
Enterotoxigenic E. coli (ETEC)	Not detected
Shiga toxin producing E. coli	Not detected
Shigella/Enteroinvasive E. coli	Not detected
Cryptosporidium species	Not detected
Cyclospora cayetanesis	Not detected
Entamoeba histolytica DNA	Not detected
Giardia lamblia DNA	Not detected
Adenovirus F40/41	Not detected
Astrovirus	Not detected
Norovirus GI/GII	Not detected
Rotavirus RNA	Not detected
Sapovirus	Not detected

**Figure 1:**
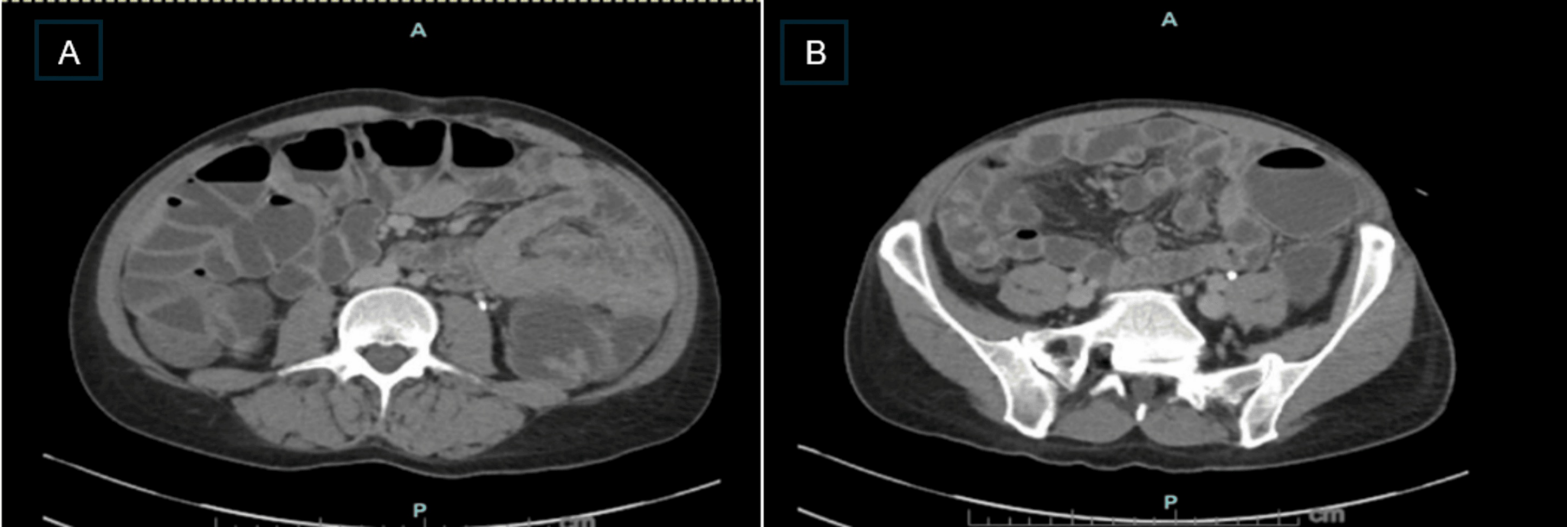
CT abdomen/pelvis imaging on presentation Axial CT images of the abdomen showing a fluid-filled, distended colon (A) and small bowel loops (B).

Computed tomography of the abdomen and pelvis demonstrated fluid-filled, distended bowel loops without toxic megacolon or perforation (Figure 1).

**Figure 2:**
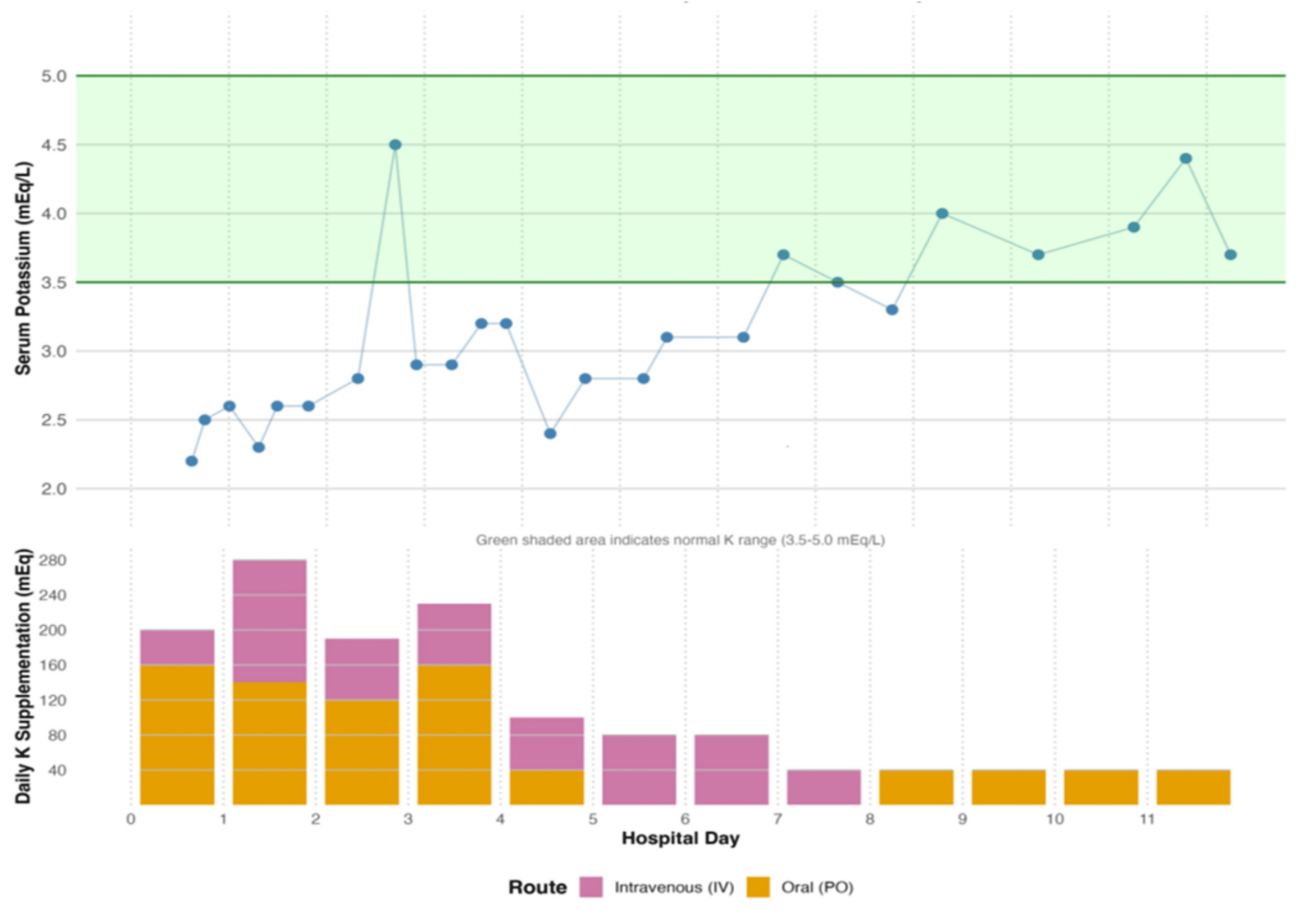
Serum potassium trend and replacement over the hospital course

Immunotherapy was immediately discontinued. Aggressive resuscitation was initiated with Ringer's lactate and potassium chloride supplementation (480 mEq in the first 48 hours). Intravenous methylprednisolone 60 mg every 12 hours was commenced for suspected ICIC. Despite these interventions, diarrheal output escalated to >10 watery bowel movements daily, with nadir potassium of 2.3 mmol/L and persistent non-anion gap metabolic acidosis. Total potassium supplementation throughout hospitalization exceeded 1,900 mEq. Serum potassium levels and potassium repletion during hospitalization is shown in Figure 2.

**Figure 3:**
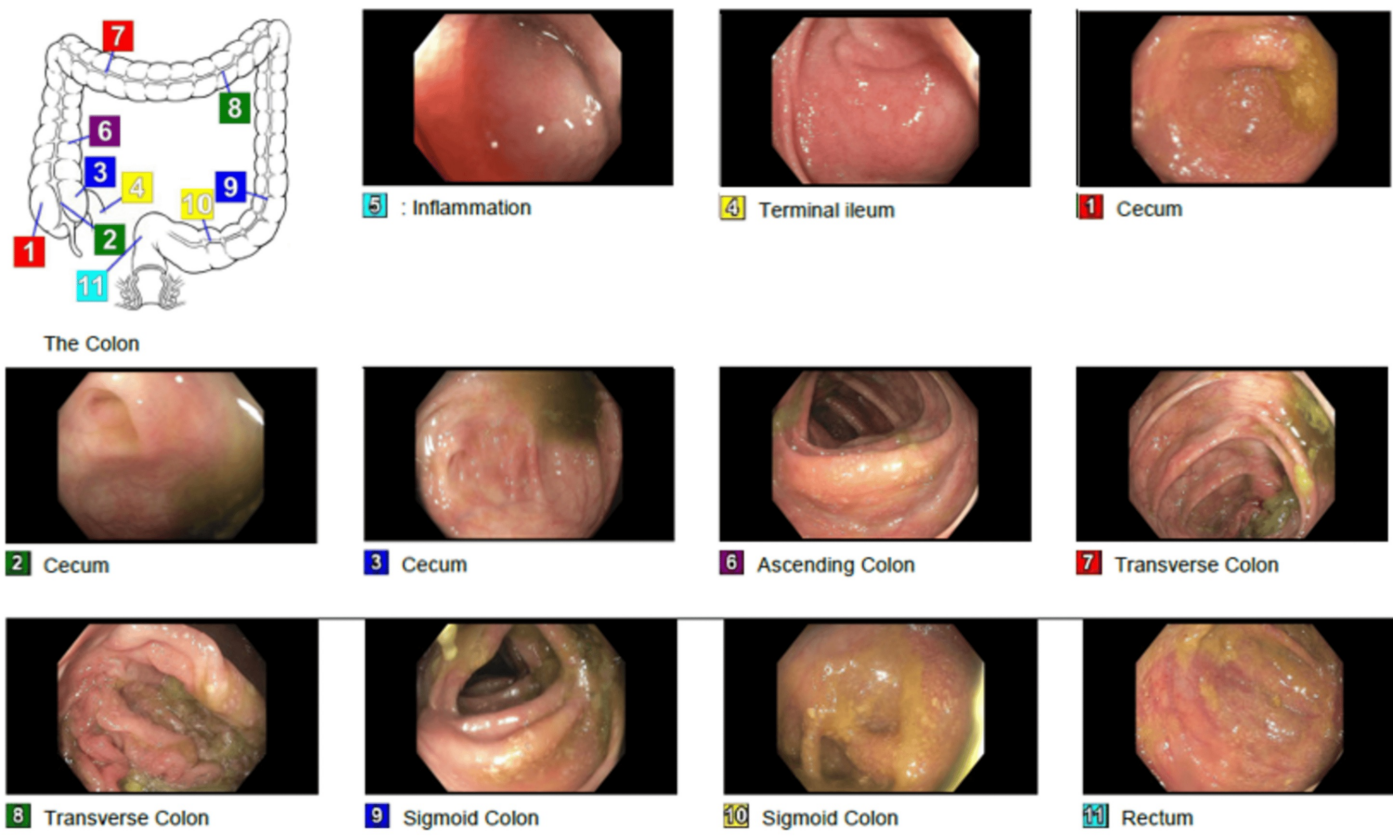
Colonoscopy done on day 4 of admission Demonstrating diffuse moderate inflammation characterized by erythema, friability, granularity, and aphthous ulceration found in the entire colon

**Figure 4:**
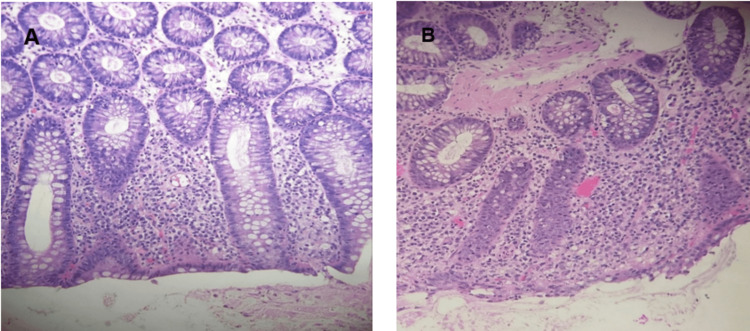
Histopathological images of biopsies taken during colonoscopy A. Colonic mucosa showing mild crypt architectural distortion with closely packed but irregularly shaped crypts and increased crypt epithelial apoptosis. B. Colonic mucosa demonstrating expansion of the lamina propria by a lymphocytic infiltrate with scattered neutrophils.

Colonoscopy on day 4 of hospitalization revealed diffuse moderate inflammation characterized by erythema, friability, and granularity throughout the entire colon (Figure 3). Histopathologic examination demonstrated mild chronic active colitis characterized by crypt architectural distortion, lamina propria lymphocytic expansion, and increased crypt apoptosis--findings consistent with ICIC (Figure 4) [5,6].

Comprehensive stool analysis revealed fecal pH <5.1, sodium 139 mmol/L, and potassium 44.3 mmol/L, yielding a calculated stool osmotic gap of approximately 123 mOsm/kg, indicating a significant osmotic component. Urine electrolyte studies demonstrated a markedly negative urine anion gap, confirming intact renal ammonium excretion and localizing the metabolic acidosis to gastrointestinal bicarbonate losses [7].

Given persistent high-volume diarrhea and bloating, empiric therapy for suspected small intestinal bacterial overgrowth was initiated with rifaximin 550 mg three times daily and probiotic supplementation [8].

Progressive clinical improvement commenced after hospital day 8, with stool frequency decreasing to approximately 4 per day, normalization of serum potassium to 4.2 mmol/L, and resolution of metabolic acidosis. The patient was discharged with a 4-week duration of oral prednisone with dose taper, completion of a 14-day rifaximin course, and coordinated follow-up.

## Discussion

This case illustrates the complex interplay among ICIC, bacterial co-infection, and suspected bacterial overgrowth, highlighting critical diagnostic and therapeutic considerations when managing severe diarrheal illness in patients receiving ICI therapy.

Pathophysiology

ICIC arises from loss of mucosal immune tolerance secondary to checkpoint blockade, resulting in activated cytotoxic T-cell-mediated injury to the intestinal epithelium [1,2]. Characteristic histopathologic findings include cryptitis, crypt abscesses, architectural distortion, and increased apoptosis--all observed in our patient (Figure 4) [5,6].

EPEC causes disease through adherence to enterocytes and effacement of microvilli, usually resulting in chloride-rich secretory diarrhea [3,4]. However, the patient had osmotic diarrhea with mild villi destruction, demonstrating that the typical laboratory values may not present when there are several factors in play.

Diagnostic approach

This case demonstrates the importance of comprehensive assessment beyond a presumptive ICIC diagnosis. Endoscopic evaluation with histopathologic confirmation remains essential for distinguishing immune-mediated from purely infectious colitis. The presence of EPEC on multiplex PCR did not exclude ICIC; conversely, histologic confirmation of ICIC did not negate the clinical significance of concurrent infection.

Stool electrolyte analysis provides critical mechanistic insights into the causes of diarrhea, but a single value shouldn't independently guide our treatment decision, as seen in our case. The markedly negative urine anion gap definitively localized the metabolic acidosis to gastrointestinal bicarbonate losses rather than renal tubular dysfunction [7].

Therapeutic considerations

Management of EPEC differs from that of Shiga toxin-producing E. coli (STEC). Because EPEC lacks Shiga toxin and the associated risk of hemolytic uremic syndrome, antibiotics can be considered in severe cases [9,10]. Rifaximin was selected for its dual activity against non-invasive diarrheagenic E. coli and empiric treatment of suspected bacterial overgrowth [8].

The profound electrolyte derangements reflected synergistic effects of inflammatory, secretory, and osmotic diarrheal mechanisms. Despite aggressive supplementation exceeding 1,900 mEq of potassium, normalization occurred only after a reduction in stool output, underscoring the importance of always treating the underlying cause of gastrointestinal losses. The concurrent QTc prolongation (568 ms) mandated maintenance of serum potassium above 4.5 mmol/L to mitigate arrhythmic risk.

Current guidelines recommend high-dose corticosteroids for grade ≥3 ICIC with escalation to biologic immunosuppression for inadequate response [1,2]. In this case, the decision to extend corticosteroid therapy before escalation was predicated on recognition of additional diarrheal contributors beyond immune-mediated inflammation alone. Clinical improvement occurred after multimodal therapy addressing all three identified pathologic processes.

Clinical implications

This case illustrates several important principles. First, the detection of an enteric pathogen should not preclude consideration of ICIC, as co-occurrence is possible and may result in synergistic severity. Second, inadequate response to standard immunosuppression should prompt evaluation for additional contributors rather than reflexive escalation to biologic agents. Third, the physiological characterization of diarrhea provides mechanistic insights that guide targeted therapy.

## Conclusions

This case demonstrates that enteropathogenic *Escherichia coli *infection and bacterial overgrowth can significantly exacerbate ICIC, resulting in severe electrolyte derangements and prolonged clinical course. Clinicians should maintain heightened suspicion for infectious co-contributors in patients with ICIC who exhibit disproportionate fluid and electrolyte losses or fail to improve with standard immunosuppression. Comprehensive diagnostic evaluation, including multiplex stool testing, endoscopic assessment, and physiological profiling, enables the identification of all contributing mechanisms and implementation of targeted multimodal therapy.
